# Relational intelligence in AI chatbots: Examining the trust–engagement-emotion–social presence model using PLS-SEM and fsQCA

**DOI:** 10.1371/journal.pone.0356073

**Published:** 2026-08-14

**Authors:** Yang Wang, Asif Ali Safeer, Yewang Zhou

**Affiliations:** Business School, Huanggang Normal University, Huanggang, China; Bangladesh Army University of Science and Technology, BANGLADESH

## Abstract

AI-driven chatbots may have emerged as the main architects for developing relationships rather than simply processing transactions. The core challenge is to understand how AI-driven chatbots can help build customer relationships. Therefore, this study investigates how AI-driven chatbot determinants, such as personalization, interactivity, and ease of use, transform customer-chatbot relationship outcomes (i.e., engagement and emotional connection) via psychological mechanisms (i.e., chatbot trust) by incorporating the moderating role of chatbot social presence in the travel industry. An online survey was used to collect responses from the Chinese tourists who regularly used AI chatbots for their trip planning and execution. This study analyzed 690 responses using PLS-SEM and fsQCA techniques. The findings revealed that AI-driven chatbot determinants, such as personalization, interactivity, and ease of use, significantly boosted chatbot trust, which in turn enhanced relational outcomes, including engagement and emotional connection. Engagement was an effective factor for improving emotional connection. In addition, chatbot social presence as a moderator significantly enhanced engagement. Finally, the fsQCA analysis revealed that personalization, chatbot ease of use, and engagement are key factors in fostering tourists’ emotional connections with AI chatbots. This study contributes to relationship theories and provides important managerial implications.

## 1. Introduction

In today’s highly dynamic digital environment with rapidly changing customer behavior, the strategic use of AI-driven chatbots has emerged as a powerful strategy for companies to build long-lasting customer relationships [1]. A recent report indicates that 55% of businesses are utilizing AI chatbots to improve their marketing strategies, while 60% of customers favor using AI chatbots over waiting for human agents, and 74% of customers opt for AI chatbots for simple inquiries, which underscores the increasing significance of AI chatbots in business interactions. Therefore, the AI chatbot market is projected to grow eightfold by 2032 [2]. AI-powered chatbots have revolutionized the travel business, with 64% of travelers expecting customized recommendations and 65% preferring chat help, setting a high standard for demonstrable service improvements. Likewise, the market size of artificial intelligence in the travel sector was estimated at $15.7 billion in 2023 and is predicted to expand to $40.0 billion by 2030 [3]. Therefore, scholars emphasized more research in this domain [4,5]. Recent statistics revealed that approximately 80% of Chinese customers used AI-based applications to manage their trip plans [6]. Likewise, Chinese customers use AI-driven chatbots (i.e., Alibaba, Tencent) for real-time support, language translation, and customized trip planning via online travel agencies’ (i.e., Ctrip, Qunar, Fliggy, Tuniu, Meituan) networks [7]. This research is being conducted on time, demonstrating that China has enormous potential in the travel and tourism business.

In the modern era, AI chatbots are progressively assuming relational roles [8]. AI chatbots interact more naturally with humans, providing quick information through question answering, personalized services, and tailoring their language to customer preferences [9,10]. They can strengthen machine-to-human relationships and serve as relational agents that may influence customer cognitive and affective responses [11]. Similarly, AI chatbots can affect customer trust and emotions in the travel industry, where decisions are contingent upon customer experience, emotions, and high-level trust, engagement, and commitment [8,12]. Despite chatbots’ high adoption rate, little is known about how AI-driven chatbot determinants, such as personalization, interactivity, and ease of use, translate customer-chatbot relationship outcomes (i.e., engagement and emotional connection) via psychological mechanisms (i.e., chatbot trust) by integrating the moderating role of chatbot social presence in the travel industry.

AI-driven chatbots play important roles in tourism and travel industries. For example, AI chatbots with improved personalization capabilities can track consumer behavior, which may help achieve management objectives [13]. Few studies have explored personalization, indicating that it can improve customer experience and satisfaction [13,14]. Its role in building customer relationships remains understudied. AI chatbot interactions support two-way communication and build relationships [15]. Interactivity is positively associated with communication quality and user satisfaction [16]. Research exploring the impact of interactivity on customer engagement and emotional connection remains scarce. Ease of use, a widely acknowledged construct [17], endorses technology adoption, but it has been inadequately positioned as a predictor of customer-chatbot relationship outcomes, such as trust, engagement, and emotional connection. This original study incorporates three core determinants of AI-driven chatbots (i.e., personalization, interactivity, and ease of use) to predict customer-chatbot relational outcomes in the travel industry.

Relationship marketing theory accentuates the importance of customer-firm relationships by improving trust, emotional connection, and satisfaction [18]. As frontline service distribution has become increasingly digitalized, specifically in travel and tourism, AI chatbots act as relational agents that can engage customers [8]. However, chatbots may encounter trust issues stemming from their lack of authenticity and empathy, which may hinder relational depth [1,11]. China’s recent outbound tourism report revealed that Chinese customers had low trust in AI-based systems [7]. Therefore, investigating chatbot trust is highly relevant in China, where cultural differences and a high level of technological acceptance may influence customer preferences and behaviors. Previous studies revealed that determinants of AI chatbots, such as personalization, interactivity, and ease of use, can improve the quality of communication, service satisfaction, virtual experiences, and customer attitudes [13,16,19]. The role of chatbot trust as a key mediator in translating the determinants of AI-driven chatbots (personalization, interactivity, and ease of use) into customer engagement and emotional connection has been inadequately addressed in the literature.

Chatbot social presence relates to the individual’s perceptions of the chatbot as a socially conscious and emotionally responding entity [20]. A chatbot with a high social presence may boost relational consequences by enhancing trust, resulting in more engaging and emotionally resonant experiences, while a chatbot with a low social presence may fail to develop emotional connections, jeopardizing relational outcomes. Previous research has shown that social presence is an important antecedent of website design and e-commerce trust, usefulness, enjoyment, and customer loyalty [21]. Similarly, chatbot social presence can dramatically improve chatbot continuous intentions, user trust, and social-oriented communication [20,22]. The moderating role of chatbot social presence in improving chatbot-customer relational outcomes, such as engagement and emotional connection, remains underexplored in the literature. This study aims to achieve the following objectives in China’s travel industry.

To determine the effects of AI chatbot determinants, such as personalization, interactivity, and ease of use, on chatbot trust in travel service environments.

To identify the direct and indirect (mediating) effects of chatbot trust on customer engagement and emotional connection.

To find out the direct and indirect (mediating) effects of engagement on emotional connection.

To assess the moderating effects of chatbot social presence on the associations between chatbot trust and engagement, as well as between chatbot trust and emotional connection.

This study aims to contribute to relationship theories from human-to-human interactions to the machine-to-human paradigm by empirically developing a comprehensive theoretical framework. This study incorporates relationship marketing, commitment-trust, and social presence theories to offer fresh insights into the strategic use of AI-driven chatbots for enhancing emotional customer trust, engagement, and emotional connection in the travel industry. The new findings provide important theoretical contributions and practical implications for developing effective service design and managing customer relationships in digital environments. The increasing use of AI in travel services, as well as the expectations for emotionally intelligent systems, underscore the importance of this topic.

## 2. Theoretical model and hypotheses development

**Fig 1:**
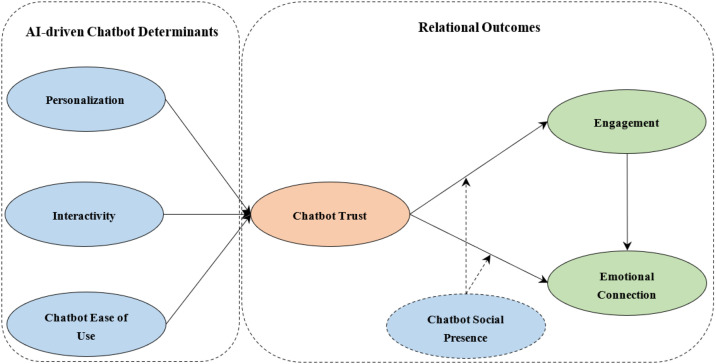
Trust–Engagement-Emotion–Social Presence (TEESP) Model: A Relational Intelligence Framework Source: “Authors’ own work”.

This study integrates relationship marketing, commitment-trust, and social presence theories to investigate the effects of AI-driven chatbot determinants like personalization, interactivity, and ease of use, on customer-chatbot relationship outcomes, such as engagement and emotional connection via chatbot trust by integrating the moderating role of chatbot social presence in the travel industry (see Fig 1).

### 2.1. AI-driven chatbot determinants (Personalization, Interactivity, and Chatbot ease of use)

Personalization involves the ability of AI chatbots to analyze content about customer needs and behaviors, providing customized recommendations that improve customer satisfaction through virtual interactions [13]. Relationship marketing theory (RMT) asserts that trust is a core factor that strengthens customer-firm relationships [18], which may foster long-term commitment, engagement, and emotional connection. Extending the RMT to AI-related travel service experiences posits that machine-human interactions can foster trust, as human-to-human interactions do in traditional service delivery. In this context, personalization serves as a crucial relational instrument through which firms can demonstrate recognition, responsiveness, and individualized value to customers, which cultivates trust [13,18]. According to RMT, trust is a key component of long-term relationships that grows over time through personalized communication that exceeds customer expectations [23]. AI-driven chatbots that create personalized content or recommendations following customers’ behavior, likes, or localized needs promote a perception of personalized attention and affection. Such personalized interactions foster relational trust and boost perceived service quality, indicating that the AI-driven chatbots are dependable and prioritize the customer’s best interests [14,24]. Therefore, personalization may boost the firm’s relational value and benevolence, which can increase trust in AI-driven chatbots. It is proposed:

H1a: Personalization significantly influences chatbot trust.

Interactivity has become a foundational concept in service quality and relationship marketing research. It can be defined as the chatbot’s ability to engage in real-time, reciprocal communication with customers while maintaining conversational consistency [16,25]. Interactivity improves the functionality of AI technology, resulting in better responses, increased trust, and greater user control over the communication process [26]. Within the context of relationship marketing theory, interactivity facilitates two-way communication and responsiveness in relational exchanges, which may assist in building customer trust [18,24]. Previous studies demonstrated that chatbot interactivity enhances communication service quality, customer satisfaction, and service co-creation [14,16,27], which may assist in developing customer trust. AI-driven chatbots mimic human-like communication, acting as relationship agents that can improve through reciprocal communication flow, fostering relationship development and mutual trust [26]. Interactive chatbots reduce cognitive load and support dialogic planning adjustments. For example, customers who customize trip planning to meet their immediate needs can improve their trust in AI chatbots. The rising expectations of Chinese customers for responsive, intelligent service agents in digital ecosystems accentuate the strategic importance of interactivity in relationship quality. According to RMT, chatbot interactions may promote customer participation and develop trust. It is proposed:

H1b: Interactivity significantly influences chatbot trust.

Ease of use refers to “the degree to which a person believes that using a particular system would be free of effort” [17, p. 320]. Chatbot ease of use can be defined as the extent to which customers believe that interacting with a chatbot would be effortless, easy, and friendly [28]. Previous studies investigated the ease of use to understand user satisfaction and chatbot usage intentions [11,28]. However, little is known about the influence of chatbot ease of use on customer trust in chatbots, particularly in the travel industry. Clear, understandable, and easy-to-use customer-chatbot interactions can assist in building initial trust in chatbots [29]. Relationship marketing theory asserts that supporting easy interactions and reducing customer effort improve the relational exchange process, enhancing customer trust and satisfaction in services where ambiguity and complexity may undermine it [23,24]. Ease of use has emerged as a key relational factor in China’s travel industry, where AI is prevalent, and tech-savvy Chinese travelers demand easy, fast, and effective interactions. As a result, chatbot ease of use can be an effective measure to build trust and relationships with customers.

H1c: Chatbot ease of use significantly influences chatbot trust.

### 2.2. Chatbot trust

Trust can be a critical factor in developing dependable, successful interactions between humans and chatbots [1]. Previous research highlighted the importance of trust for developing customer interactions with AI chatbots [29]. However, prior research has paid little attention to investigating customer trust in AI chatbots. Previous studies have focused on evaluating the effects of chatbot trust on user intentions to use chatbots and e-brand loyalty [29–31]. According to commitment-trust theory (CTT), trust serves as the bedrock of long-lasting relationship interactions [18]. Previous research indicates that CTT plays an important role in enhancing consumer-brand interactions and other key stakeholders in the social media context [32]. Therefore, chatbot trust may reduce perceived risk and uncertainty, which can motivate customers to interact with chatbots more frequently. Similarly, relationship marketing theory supports that when customers feel relational trust, they reciprocate with proactive behavior, including interactive questions, co-creation, and service advocacy [24]. Chinese travel platforms offer a wide range of service quality choices, and travelers can rely on relational cues to assess chatbot trust. As a result, when chatbots respond quickly, precisely, and empathically, customers may be more inclined to interact with them more deeply. It is proposed:

H2a: Chatbot trust significantly influences engagement.

RMT posits that personalized content and recommendations can indicate relational value and responsiveness, reflecting care and trust at the individual level [23,24]. Interactivity accentuates two-way communication through real-time conversation and receptive follow-ups [16]. It represents relational responsiveness, which can increase trust. CTT postulates that such responsive communication decreases perceived risk and uncertainty and fosters trust in the service agent over time [18]. Chatbot ease of use signifies simple service accessibility and decreases relational challenges, indicating care for customers’ comfort, which can enhance trust and satisfaction [28]. Previous studies have shown that personalization and interactivity can positively influence user satisfaction through communication quality and the virtual flow experience [13,16]. Likewise, research revealed that ease of use can enhance actual use of AI chatbots by shaping users’ intentions to use chatbots [11]. However, research examining the determinants of AI-driven chatbots, such as personalization, interactivity, and ease of use on customer engagement via chatbot trust, particularly in the Chinese travel industry, remains scarce. Therefore, this study intends to offer new insights into literature. It is proposed:

H2b: Chatbot trust positively mediates the relationship between personalization and engagement.

H2c: Chatbot trust positively mediates the relationship between interactivity and engagement.

H2d: Chatbot trust positively mediates the relationship between chatbot ease of use and engagement.

Brand success in the service sector, specifically travel, depends heavily on building trust and cultivating long-term customer relationships. CTT suggests that trust is a key antecedent of relational exchanges and emotional attachment with a brand [18]. RMT postulates that trust develops through continued value-generating relationships and provides the basis for attachment, which can boost emotional connection [23,24]. These theories provide a strong foundation for understanding how AI-driven chatbot trust influences customers’ relational and emotional responses, including emotional connection. Emotional connection refers to the feelings that customers experience when they are emotionally attached to a brand [33]. This study defines emotional connection as a customer’s sense of psychological empathy, intimacy, and emotional association with the AI chatbot. AI chatbots are often characterized as AI companions for their ability to foster relational closeness and empathy, which enhances deeper relationships with users [34]. RMT emphasizes that effective relationships extend beyond transactional interactions, integrating trust and emotional connections to strengthen relational strength [18]. Previous studies have emphasized the outcomes, such as chatbot usage intentions and customer satisfaction [13,16,19]. Research focusing on the associations between AI chatbot trust and customer emotional connection is rare. It is hypothesized:

H3a: Chatbot trust significantly influences emotional connection.

AI chatbot determinants, such as personalization, interactivity, and ease of use, may serve as building blocks for developing emotional connections with customers via trust. Relationship marketing and trust commitment theories support the notion that favorable relationships enhance trust, resulting in higher customer attachment to the brand [18,23,24]. Previous studies have revealed that personalization, interactivity, and ease of use can improve customer satisfaction and their intentions to use chatbots [11,13,28]. These factors may motivate customers to build trust in chatbots [29,31], potentially leading to greater attachment to chatbots [1]. In the Chinese travel industry, where AI implementation and adoption rates are rapid and customer digital literacy is high, personalization, interactivity, and ease of use can be factors that influence customer trust in AI travel chatbots, which may deepen their relationship with chatbots. In line with RMT and CTT, it can be proposed:

H3b: Chatbot trust positively mediates the relationship between personalization and emotional connection.

H3c: Chatbot trust positively mediates the relationship between interactivity and emotional connection.

H3d: Chatbot trust positively mediates the relationship between chatbot ease of use and emotional connection.

### 2.3. Engagement and emotional Connection

RMT posits that engagement is imperative for developing relational mechanisms, which connect transactional exchanges and long-term relationships [24]. Previous studies have argued that engagement serves as a key component of relationships and contributes to customer satisfaction, purchase intentions, and price premium [29,35,36]. Research investigating the impact of engagement on emotional connection with AI chatbots in the Chinese travel industry remains untapped. CTT reiterates its argument by demonstrating that consumers are prone to form emotional relationships when they engage in relational commitments, as reflected in their behaviors [18]. In the Chinese travel industry, where AI-driven travel chatbots interact with different customers, engagement is important for building relationships through information exchange and continuous collaborative dialogues, which may foster customer emotional resonance. In line with RMT and CTT, it is hypothesized:

H4a: Engagement significantly influences emotional connection.

Service relationship models anchored in RMT and CTT emphasize that trust is an integral component in fostering deeper cognitive and affective relationships [18,24]. The proposed hypothesis might extend relationship theories by demonstrating that engagement positively impacts the relationship between chatbot trust and emotional connection in contemporary AI-driven travel chatbot contexts. Previous research supports the premise that trust can enhance customer engagement [29], and engagement may foster customer satisfaction [36]. Emotional connections are more likely to form when customers trust AI chatbots, which may lead to meaningful relationships between customers and AI chatbots. Therefore, engagement can be a central construct that meaningfully translates trust into emotional connections. It is hypothesized:

H4b: Engagement positively mediates the relationship between chatbot trust and emotional connection.

### 2.4. Chatbot social presence

Social presence refers to the degree to which a channel facilitates individuals to experience other individuals as psychologically present [21]. Social presence theory [37] was historically applied to human-to-human interactions, but its importance has grown in the context of AI-human communication. This study postulates that chatbots with a high social presence, such as warm, anthropomorphic, and empathetic, as well as socially driven conversational clues, foster relational significance of trust and behavioral consequences. Social presence boosts parasocial interaction, perceived dialogue quality, and psychological involvement, which in turn may increase engagement [38]. CTT suggests that trust acts as a cognitive driver for relational behavior and engagement [18], but without relational signals, trust may be considered as utilitarian or abstract. Prior studies have emphasized the role of chatbot social presence to understand users’ continuous intentions, trust, and social-oriented communication [20,22]. However, research on the moderating effects of chatbot social presence is rare. With the support of CTT and social presence theory, it hypothesized:

H5a: Chatbot social presence positively moderates the relationship between chatbot trust and engagement; trust has a greater effect on engagement when chatbot social presence is high and vice versa.

RMT highlights that relationship warmth and commitment are vital for cultivating a deeper emotional attachment [23]. Additionally, CTT demonstrates that trust is a key factor for developing relationships; hence, social cues enhance its impact by reinforcing relational value [18]. Emotional connection can be described as a feeling of psychological intimacy, love, and affective relationships with the AI chatbot, which serves as a brand ambassador [39]. Social presence theory posits the significance of interactive communication across different media in conveying verbal and visual information, and for influencing individuals’ ability to communicate their perceptions of others’ physical presence [40]. Previous studies have investigated the role of social presence in shaping dialogues and parasocial interactions with chatbots, and chatbot continuous intentions [20,38]. Previous research supports the argument that continuous dialogues and parasocial interactions may enhance closeness, love, and affective relationships with chatbots [38,39]. Therefore, it can be proposed:

H5b: Chatbot social presence positively moderates the relationship between chatbot trust and emotional connection; trust has a greater effect on emotional connection when chatbot social presence is high and vice versa.

## 3. Materials and methods

This study was designed to collect data via an online survey from tourists who used AI-driven chatbots for trip planning and execution while traveling to different cities in China. The survey methodology aligns with previous research, indicating that it’s an effective methodology to understand customer behavior and complex relationships in a technology-mediated environment [13,28,41]. The survey included the following screening questions to identify true respondents:

- Have you planned or booked a trip within the last 6 months? Yes/ No
- Have you used an AI-powered chatbot (e.g., WeChat, Alipay, Meituan, Qunar, Fliggy, Dianping) for tourism-related services (e.g., trip planning, booking)? Yes/ No
- How would you rate your familiarity with technology?
- Very Low
- Low
- Moderate
- High
- Very High

The original survey was developed in English and subsequently translated into simplified Chinese by a native Chinese professor through a rigorous translation process.

### 3.1. Sampling and data collection procedures

This study targeted Chinese adult tourists who primarily interacted with travel-related AI chatbots, such as Fliggy, Ctrip, Dianping, WeChat, Alipay, Meituan, Qunar, and trip.com. These digital platforms are China’s leading travel service providers, offering real-time trip-planning information and active customer care via AI chatbots. Consistent with previous research, this study used purposive sampling to collect data from respondents [42]. Purposive sampling was deemed suitable to verify that participants had adequate knowledge and experience to effectively assess the study constructs. The respondents were recruited by sharing an online survey QR code via WeChat (a leading platform) and contacting them personally. The survey was posted in Chinese on a leading survey platform (i.e., www.wjx.cn) from February 2, 2025, to March 3, 2025. Respondents had to rate each question on a scale of seven points. For example, they had to provide a higher rating (i.e., 7) to questions on which they were extremely in agreement and a lower rating (i.e., 1) to those on which they were extremely in disagreement. The respondents who completed the survey were rewarded with 2 yuan as a token of appreciation. The modest financial incentive helps reduce potential risks and fosters respondents’ engagement in providing careful responses to survey questions. Each participant was allowed to respond to the survey once. We applied several measures to enhance data quality, such as IP address tracking to prevent duplicate submissions, screening questions to confirm eligibility, and removing straight-lined responses. This study strictly adhered to ethical principles about the confidentiality and anonymity of respondents’ data.

Initially, 90 respondents were recruited for a pretest to evaluate tourists’ perceptions of the survey questions and to validate the scales. After data screening, 80 responses were considered for pretest analysis. The results revealed that all outer loading values were greater than 0.70, except for chatbot trust item 2 (i.e., 0.65). Likewise, Cronbach’s alpha and composite reliability values exceeded 0.70, and convergent validity (AVE) values surpassed 0.50, demonstrating the reliability and validity of the survey items [43]. After receiving feedback from some respondents, a few initial check questions were revised, and the survey was distributed to the mass population, resulting in 750 new responses received. After data filtration, 690 responses were included in the analysis. Table 1 illustrates the demographic information of the respondents. This study mainly emphasized the younger demographic (i.e., aged 18–33 years) because they are technology-savvy, better educated, and an important market segment for many companies [10,41,44]. The data was accessed on March 4, 2025, for analysis purposes. However, the authors did not have access to identifying information about respondents.

**Table 1:** Respondent demographic information.

Description	Number	%
**Sample size**	**690**	100.00%
**Gender**		
Male	342	49.57%
Female	348	50.43%
**Age**		
18–25	317	45.94%
26–33	194	28.12%
34–41	75	10.87%
42–49	60	8.70%
Above 50	44	6.38%
**Education**		
High School	163	23.62%
Undergraduate	259	37.54%
Graduate	165	23.91%
Post-graduate	103	14.93%
**Occupation**		
Student	221	32.03%
Private Firm Employee	250	36.23%
Government employee	91	13.19%
Entrepreneurs’	51	7.39%
Unemployed	77	11.16%

Source: “Authors’ own work”

This study involves human participation and was approved under Ref. No. HGNU/ERC/25/0102 dated 2025-01-10 by the Ethical Review Committee at the Business School, Huanggang Normal University, Huanggang, China. This study was conducted in accordance with the local legislation and institutional requirements. The participants were provided with written informed, voluntary consent to participate in this study. The minors were excluded from participating in the study. Participants had the option to withdraw from the survey at any time during its completion.

### 3.2. Measures

This study measured 25 items to discover novel insights in a relational intelligence framework (See Fig 1). The scales were adjusted from previous research published in top-tier journals. The determinants of AI-driven chatbots, such as personalization and interactivity, were measured using three items each, and chatbot ease of use was measured with four items [13,16,45]. Relational outcomes, such as chatbot trust and emotional connection, were assessed using three items each, and engagement was assessed with four items [29,33,46]. Chatbot social presence was assessed with five items [21].

### 3.3. Common method bias

It is critical to assess data bias, as survey methodologies may induce self-selection bias. This study employed two established tests to identify bias in the data. First, we performed Harman’s single-factor as a diagnostic test for bias [47,48]. The findings indicated that a single factor accounted for 48.95% of the total variance, which was below the 50% threshold, suggesting that the bias does not pose a significant threat to the data. However, the Harman single factor test has limitations and is inadequate for demonstrating the absence of bias in the data. Therefore, we employed a full collinearity test using the variance inflation factor (VIF) [49]. The findings revealed that all VIF values are below the threshold of 3.30, indicating no potential threat of bias in the data (see Table 2).

**Table 2:** Construct reliability, convergent validity, and collinearity statistics (VIF).

Code	Item Description	Loading	VIF
**AI-driven Chatbot Determinants**			
**Personalization (α = 0.886; CR (rho_c) = 0.886; CR (rho_A) = 0.892; AVE = 0.723)**			
PRS1	AI-driven chatbots provide tailored information about travel and tourist places.	0.929	2.544
PRS2	I can obtain customized information about travel and tourism by interacting with AI-driven chatbots.	0.789	2.612
PRS3	The personalized information for travel and tourism offered by AI-driven chatbots satisfies my needs.	0.827	2.436
**Interactivity (α = 0.857; CR (rho_c) = 0.857; CR (rho_A) = 0.857; AVE = 0.666)**			
INT1	I can find many other tourists’ questions and responses on travel and tourism by using AI-driven chatbots.	0.797	1.951
INT2	The AI-driven travel and tourism chatbots that I use are extremely responsive to users.	0.828	2.368
INT3	It is easy to share travel and tourism information using AI-driven chatbots.	0.824	2.213
**Chatbot Ease of Use (α = 0.902; CR (rho_c) = 0.901; CR (rho_A) = 904; AVE = 0.696)**			
CEU1	My interactions with AI-driven chatbots are clear and understandable.	0.805	2.657
CEU2	AI-driven chatbots are easy to use in my opinion.	0.833	2.696
CEU3	Interacting with AI-driven chatbots requires less mental effort.	0.912	2.328
CEU4	I find it easier to achieve the AI-driven chatbots to perform what I want them to.	0.779	2.652
**Relational Aspects of AI Chatbots**			
**Chatbot Trust (α = 0.822; CR (rho_c) = 0.821; CR (rho_A) = 0.822; AVE = 0.605)**			
CBT1	AI-driven chatbots look reliable.	0.803	1.788
CBT2	AI-driven chatbots are designed to assist the user.	0.771	1.907
CBT3	AI-driven chatbots appear trustworthy.	0.760	1.840
**Engagement (α = 0.879; CR (rho_c) = 0.879; CR (rho_A) = 0.879; AVE = 0.644)**			
ENG1	Using AI-driven chatbots is interesting.	0.806	2.142
ENG2	Using AI-driven chatbots captures my attention.	0.814	2.139
ENG3	Using AI-driven chatbots is engaging.	0.804	2.294
ENG4	I am totally involved in using AI-driven chatbots.	0.785	2.215
**Emotional Connection (α = 0.820; CR (rho_c) = 0.820; CR (rho_A) = 0.821; AVE = 0.603)**			
EMC1	I am emotionally attached to AI-driven chatbots.	0.800	1.869
EMC2	I am emotionally bonded with AI-driven chatbots.	0.769	1.900
EMC3	I am emotionally connected with AI-driven chatbots.	0.762	1.747
**Chatbot Social Presence (α = 0.883; CR (rho_c) = 0.883; CR (rho_A) = 0.884; AVE = 0.602)**			
CSP1	AI-driven chatbots provide a sense of human touch.	0.806	2.149
CSP2	AI-driven chatbots possess a feeling of personalness.	0.768	1.894
CSP3	AI-driven chatbots have a sociable nature.	0.791	2.218
CSP4	AI-driven chatbots exhibit human warmth.	0.741	2.079
CSP5	AI-driven chatbots exhibit human-like sensitivity.	0.770	2.075

Source: “Authors’ own work”

Assessing endogeneity is essential since its absence shows that the data is unbiased, ensuring the validity of the findings and their significant contribution to literature [43]. The endogeneity issue arises when exogenous variables are linked to the error term of endogenous variables. We used the Gaussian copula approach to evaluate the potential existence of endogeneity [50]. The results indicate that all Gaussian copula conditions are statistically non-significant (p > 0.05), suggesting that endogeneity does not pose a serious threat to the structural model relationships [50,51]. As a result, the findings validate the robustness and reliability of the structural model estimates.

## 4. Results

This study employed symmetric (PLS-SEM) and asymmetric (fsQCA) analyses to reveal fresh insights. Symmetric analysis can be performed through PLS-SEM. PLS-SEM measures the proposed theoretical model in two phases: measurement model evaluation and structural model evaluation [43]. We applied the PLSc algorithm to evaluate the measurement and structural model [52].

### 4.1. Measurement model evaluation

Measurement of the outer model evaluation is critical for the constructs’ reliability and validity. Current literature demonstrates that scholars must follow standard procedures to ensure the robustness of the measurement model. Therefore, robust criteria for indicator loadings, Cronbach’s alpha, composite reliability, AVE, and HTMT must be followed to ensure the accurate reliability and validity of the proposed model [43,53]. Table 2 shows that all the values of internal consistency reliability, such as indicator loading, Cronbach’s alpha, and composite reliability, are greater than 0.70, and convergent validity (i.e., AVE) values surpassing 0.50, demonstrating that the measurement model has satisfied the threshold [43]. The findings revealed that all constructs, including AI chatbot determinants (personalization, interactivity, chatbot ease of use) and AI chatbot relational aspects (chatbot trust, engagement, emotional connection, and chatbot social presence), demonstrating construct reliability and validity.

Discriminant validity was calculated using the well-renowned HTMT ratio criterion [53]. Table 3 shows that all HTMT values are below 0.85, indicating that the measurement model has achieved sufficient discriminant validity [53]. The findings demonstrated that AI chatbot determinants (personalization, interactivity, and chatbot ease of use) and AI chatbot relational aspects (chatbot trust, engagement, emotional connection, and chatbot social presence) are significantly distinct from each other, thereby validating discriminant validity. Furthermore, we assessed discriminant validity by analyzing item cross-loadings. Table 4 indicates that all items exhibit the highest loading values on their corresponding constructs, which consistently exceed the cross-loading values of other constructs. These findings demonstrate that each construct effectively represents a unique concept, which supports discriminant validity [43].

**Table 3:** Discriminant validity; Heterotrait-monotrait ratio.

Construct	1	2	3	4	5	6	7
1. Chatbot Ease of Use							
2. Chatbot Social Presence	0.679						
3. Chatbot Trust	0.678	0.835					
4. Emotional Connection	0.599	0.786	0.843				
5. Engagement	0.570	0.743	0.818	0.826			
6. Interactivity	0.655	0.707	0.678	0.614	0.585		
7. Personalization	0.598	0.720	0.708	0.624	0.586	0.600	

Source: “Authors’ own work”

**Table 4:** Cross loadings.

Items	CBT	CEU	CSP	EMC	ENG	INT	PRS
CBT1	**0.803**	0.512	0.672	0.689	0.671	0.556	0.563
CBT2	**0.771**	0.542	0.631	0.659	0.631	0.514	0.526
CBT3	**0.760**	0.533	0.648	0.621	0.607	0.512	0.565
CEU1	0.547	**0.805**	0.537	0.474	0.450	0.514	0.485
CEU2	0.566	**0.833**	0.563	0.492	0.466	0.539	0.461
CEU3	0.620	**0.912**	0.636	0.550	0.534	0.606	0.547
CEU4	0.529	**0.779**	0.532	0.486	0.456	0.528	0.501
CSP1	0.706	0.580	**0.806**	0.624	0.609	0.566	0.568
CSP2	0.646	0.508	**0.768**	0.598	0.576	0.552	0.567
CSP3	0.650	0.519	**0.791**	0.622	0.588	0.540	0.564
CSP4	0.612	0.534	**0.741**	0.582	0.552	0.555	0.539
CSP5	0.626	0.500	**0.770**	0.620	0.557	0.531	0.552
EMC1	0.683	0.500	0.625	**0.800**	0.656	0.504	0.499
EMC2	0.634	0.465	0.613	**0.769**	0.639	0.491	0.499
EMC3	0.649	0.434	0.591	**0.762**	0.630	0.436	0.455
ENG1	0.645	0.469	0.607	0.674	**0.806**	0.436	0.481
ENG2	0.676	0.488	0.609	0.660	**0.814**	0.487	0.497
ENG3	0.665	0.452	0.595	0.662	**0.804**	0.500	0.467
ENG4	0.641	0.428	0.576	0.657	**0.785**	0.454	0.440
INT1	0.541	0.522	0.531	0.475	0.444	**0.797**	0.471
INT2	0.561	0.545	0.577	0.515	0.468	**0.828**	0.487
INT3	0.559	0.542	0.623	0.514	0.519	**0.824**	0.500
PRS1	0.658	0.528	0.652	0.572	0.550	0.463	**0.929**
PRS2	0.559	0.485	0.579	0.509	0.470	0.517	**0.789**
PRS3	0.586	0.514	0.602	0.509	0.474	0.548	**0.827**

Source: “Authors’ own work”

### 4.2. Structural model evaluation

**Fig 2:**
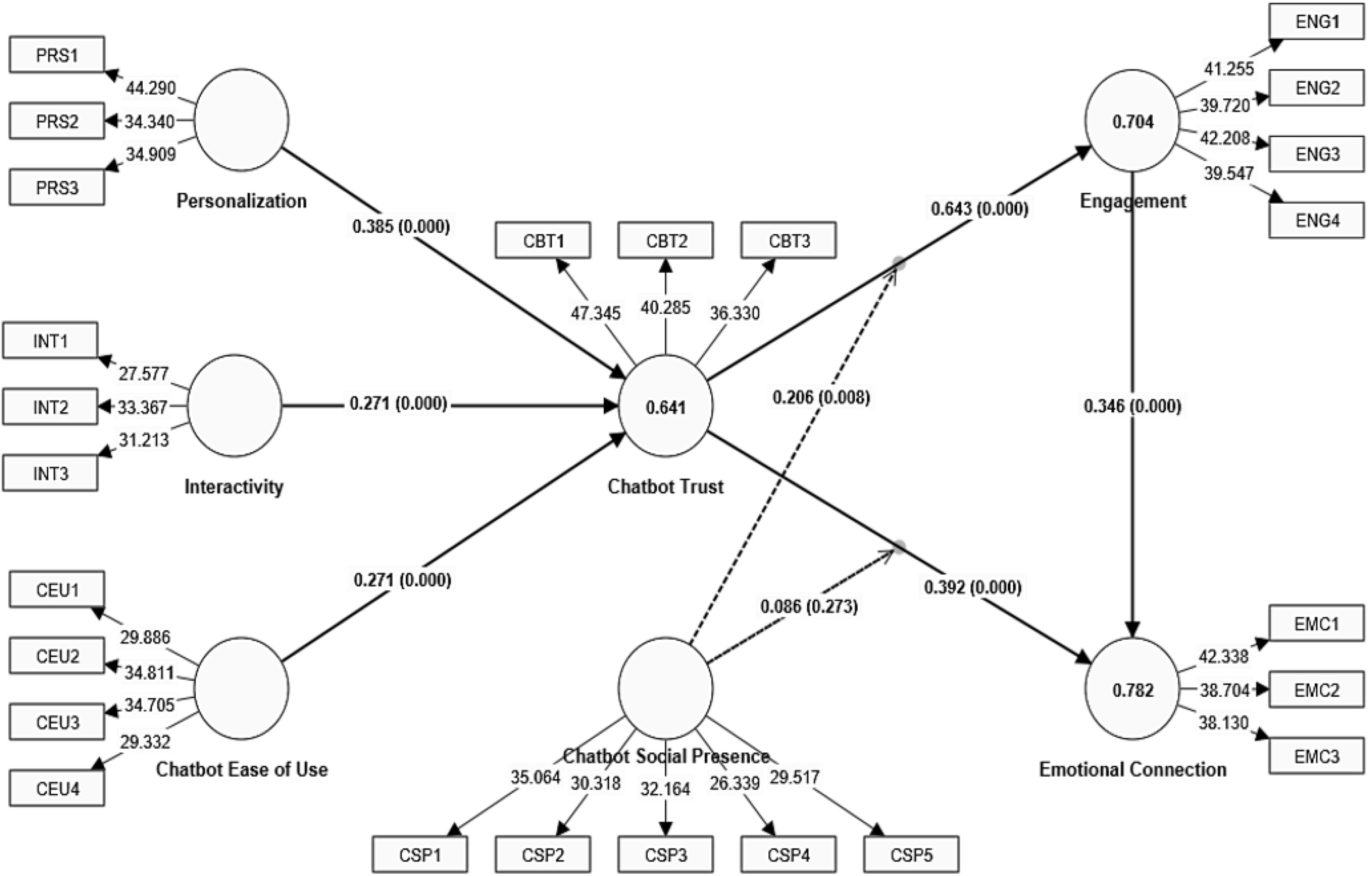
Structural Model Results Source: “Authors’ own work”.

The structural model evaluates the inner model. It examines multicollinearity, R^2^, model fit, Q^2^, effect size, and the relationships of proposed hypotheses [43]. First, we examined multicollinearity using the variance inflation factor (VIF) metric. Table 2 shows that all VIF values are below 3, demonstrating that the data are free from multicollinearity and bias [54]. Second, the model’s explanatory power was assessed via the R^2^ metric. Fig 2 illustrates R^2^ values of 0.64 for chatbot trust, 0.70 for engagement, and 0.78 for emotional connection, demonstrating that the proposed model sufficiently explains the variance in all endogenous constructs [55]. Third, model fit was determined through SRMR metrics. The results showed that the SRMR value was 0.05, which is less than 0.08, explaining an excellent model fit [54]. Fourth, the model’s predictive relevance (Q^2^) was calculated using blindfolding procedures. The results showed that Q^2^ values of 0.36 for chatbot trust, 0.39 for engagement, and 0.44 for emotional connection, demonstrating sufficient predictive relevance of the proposed model [43]. The effect size (*f²*) was estimated to assess the practical significance of structural interactions [43]. Research indicates that *f*^*2*^ values of 0.35, 0.15, and 0.02 correspond to large, moderate, and small effects, respectively [56]. The study found (see Table 6) that personalization had a moderate influence on chatbot trust (*f²* = 0.235), whereas interactivity and ease of use had small effects (*f²* = 0.103). Chatbot trust had strong effects on engagement (*f²* = 0.420) but moderate effects on emotional connection (*f²* = 0.149). Likewise, engagement had a moderate effect on emotional connection (*f²* = 0.163). Chatbot social presence had a small but meaningful moderating effects on the association between chatbot trust and engagement (*f²* = 0.078) [43,57], but little impact on the relationship between chatbot trust and emotional connection (*f²* = 0.017).

This study applied bias-corrected accelerated bootstrap procedures with 10,000 subsamples (two-tailed, α = 0.05) to estimate path models and test hypotheses [43]. Table 5 presents a sequential path analysis that includes various model types and combinations to validate the findings. Model 1 shows the impact of AI chatbot determinants, such as personalization, interactivity, and ease of use, on trust in AI chatbots. Model 2 demonstrates the impact of AI chatbot determinants, such as personalization, interactivity, and ease of use, on user engagement and emotional connection. Model 3 presents results with mediation, whereas Model 4 includes comprehensive results, outlining direct, indirect, and interactive effects. These findings reinforce the validation of the results. Table 6 and Fig 2 explicitly demonstrate the structural model results of hypothesized relationships. The findings revealed that AI-driven chatbot determinants, such as personalization, interactivity, and chatbot ease of use, significantly improved chatbot trust, supporting the H1a-1c. Likewise, chatbot trust significantly enhanced engagement and emotional connection. Therefore, H2a and H3a were supported. The findings also revealed the positive effects of engagement on emotional connection, supporting H4a.

**Table 5:** Sequential path analysis.

Construct Relationships	Model – 1	Model – 2	Model – 3	Model – 4
**β Value**	**β Value**	**β Value**	**β Value**
Chatbot Ease of Use - > Chatbot Trust	0.272***	_____	0.271***	0.271***
Interactivity - > Chatbot Trust	0.270***	_____	0.270***	0.270***
Personalization - > Chatbot Trust	0.386***	_____	0.385***	0.385***
Chatbot Ease of Use - > Engagement	_____	0.222***	0.012(ns)	0.031(ns)
Interactivity - > Engagement	_____	0.262***	0.052(ns)	0.058(ns)
Personalization - > Engagement	_____	0.299***	0.001(ns)	−0.027(ns)
Chatbot Ease of Use - > Emotional Connection	_____	0.228***	0.017(ns)	0.015(ns)
Interactivity - > Emotional Connection	_____	0.270***	0.043(ns)	0.029(ns)
Personalization - > Emotional Connection	_____	0.326***	0.032(ns)	0.001(ns)
Chatbot Trust - > Emotional Connection	_____	_____	0.450***	0.380***
Chatbot Trust - > Engagement	_____	_____	0.774***	0.623***
Engagement - > Emotional Connection	_____	_____	0.404***	0.342***
Chatbot Ease of Use - > Chatbot Trust - > Emotional Connection	_____	_____	0.122**	0.103*
Interactivity - > Chatbot Trust - > Emotional Connection	_____	_____	0.122**	0.103**
Personalization - > Chatbot Trust - > Emotional Connection	_____	_____	0.173***	0.146**
Chatbot Ease of Use - > Chatbot Trust - > Engagement	_____	_____	0.210***	0.169***
Interactivity - > Chatbot Trust - > Engagement	_____	_____	0.209***	0.168***
Personalization - > Chatbot Trust - > Engagement	_____	_____	0.298***	0.240***
Chatbot Trust - > Engagement - > Emotional Connection	_____	_____	0.313***	0.213**
Chatbot Social Presence x Chatbot Trust - > Engagement	_____	_____	_____	0.224**
Chatbot Social Presence x Chatbot Trust - > Emotional Connection	_____	_____	_____	0.097(ns)
				
**Dependent Construct**	**R**^**2**^ **Value**	**R**^**2**^ **Value**	**R**^**2**^ **Value**	**R**^**2**^ **Value**
Chatbot Trust	0.641	_____	0.640	0.640
Engagement	_____	0.456	0.671	0.707
Emotional Connection	_____	0.507	0.770	0.783

Note: “(t > 1.96 at *p < 0.05), (t > 2.58 at **p < 0.01), (t > 3.29 at ***p < 0.001), (t<1.96 at p>0.05 (ns) non-significant), two-tailed and significance level 0.05” Source: “Authors’ own work”

**Table 6:** Hypothesis assessment results.

Hypothesis	Relationships	β value	2.50%	97.50%	t-value	*f* ^2^	Support
**Direct Effects**							
H1a	Personalization - > Chatbot Trust	0.385***	0.291	0.477	8.140	0.235	Yes
H1b	Interactivity - > Chatbot Trust	0.271***	0.176	0.369	5.461	0.103	Yes
H1c	Chatbot Ease of Use - > Chatbot Trust	0.271***	0.173	0.372	5.366	0.103	Yes
H2a	Chatbot Trust - > Engagement	0.643***	0.442	0.819	6.680	0.420	Yes
H3a	Chatbot Trust - > Emotional Connection	0.392***	0.19	0.603	3.711	0.149	Yes
H4a	Engagement - > Emotional Connection	0.346***	0.167	0.516	3.853	0.163	Yes
**Moderating Effects**							
H5a	Chatbot Social Presence x Chatbot Trust - > Engagement	0.206**	0.072	0.375	2.660	0.078	Yes
H5b	Chatbot Social Presence x Chatbot Trust - > Emotional Connection	0.086 (ns)	−0.058	0.251	1.095	0.017	No

Note: “(t > 2.58 at **p < 0.01), (t > 3.29 at ***p < 0.001), (t<1.96 at p>0.05 (ns) non-significant), two-tailed and significance level 0.05” Source: “Authors’ own work”

We conducted an analysis using bias-corrected accelerated bootstrapping procedures with 10,000 subsamples on the proposed model [43]. We identified the mediation effects by assessing the significance of indirect effects [58,59]. Table 7 presents the significant indirect effects of personalization (β = 0.247, p < 0.001; β = 0.151, p < 0.001), interactivity (β = 0.174, p < 0.001; β = 0.106, p < 0.01), and chatbot ease of use (β = 0.174, p < 0.001; β = 0.106, p < 0.01) on engagement and emotional connection, respectively, thereby supporting H2b-2d and H3b-3d. In contrast, engagement was identified as a complementary mediator (β = 0.222, p < 0.001), thus supporting H4b. These findings indicate that trust in chatbots is highly essential, while engagement serves as a significant mediator in enhancing relationships among tourists in the Chinese travel industry.

**Table 7:** Mediation Results.

Hyp.	Relationships	β value (Indirect)	2.50%	97.50%	t-value	Support	Mediation
**Mediating Effects**							
H2b	Personalization - > Chatbot Trust - > Engagement	0.247***	0.158	0.344	5.227	Yes	Indirect-only mediation
H2c	Interactivity - > Chatbot Trust - > Engagement	0.174***	0.098	0.262	4.133	Yes	Indirect-only mediation
H2d	Chatbot Ease of Use - > Chatbot Trust - > Engagement	0.174***	0.097	0.260	4.189	Yes	Indirect-only mediation
H3b	Personalization - > Chatbot Trust - > Emotional Connection	0.151***	0.072	0.240	3.523	Yes	Indirect-only mediation
H3c	Interactivity - > Chatbot Trust - > Emotional Connection	0.106**	0.046	0.187	2.939	Yes	Indirect-only mediation
H3d	Chatbot Ease of Use - > Chatbot Trust - > Emotional Connection	0.106**	0.042	0.190	2.808	Yes	Indirect-only mediation
H4b	Chatbot Trust - > Engagement - > Emotional Connection	0.222***	0.097	0.360	3.309	Yes	Complementary mediation

Note: “(t > 2.58 at **p < 0.01), (t > 3.29 at ***p < 0.001), (t<1.96 at p>0.05 (ns) non-significant), two-tailed and significance level 0.05” Source: “Authors’ own work”

**Fig 3:**
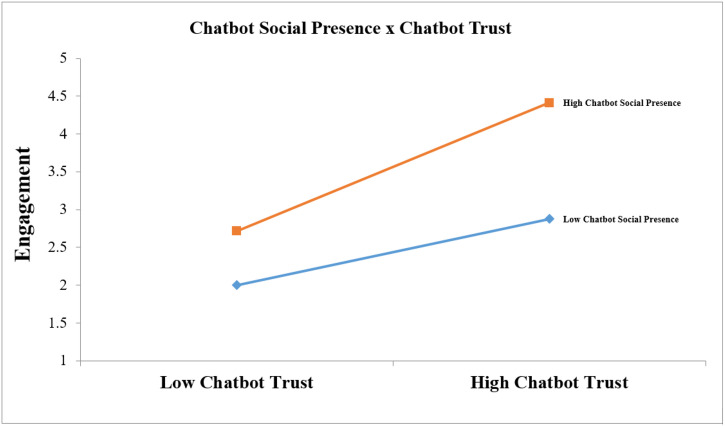
Moderation Results Source: “Authors’ own work”.

The moderating results were calculated using the interactive effects [43,54]. The findings revealed that chatbot social presence x chatbot trust significantly boosted engagement, supporting H5a as (β = 0.206, p < 0.01). Fig 3 shows that higher chatbot social presence and trust significantly enhances tourist engagement toward AI chatbots. In contrast, the interactive effects of chatbot social presence x chatbot trust did not influence emotional connection, which did not support H5b (β = 0.086, p > 0.05).

### 4.3. Asymmetric analysis (fsQCA)

Symmetric (PLS-SEM) analysis evaluates the effects of exogenous variables on endogenous variables [43]. Asymmetric analysis is based on causation, with different combinations of factors contributing to the outcomes, and fsQCA (Fuzzy Set Qualitative Comparative Analysis) is specifically designed to demonstrate equifinal, conjunctural, and asymmetric causation [60]. Previous research shows that fsQCA is important for theoretical advancement and is appropriate for large samples and regression-based frameworks [61].

#### 4.3.1. Calibration.

The fsQCA calibrates set-membership scores (e.g., 0 and 1; full non-membership fuzzy score = 0.05; crossover point fuzzy score = 0.50; and full membership fuzzy score = 0.95) and employs set-theoretic truth-table logic to derive conclusions from configural conditions. It analyzes necessary and sufficient conditions, such as frequency cut-offs, consistency thresholds, and calibration anchors [62]. We used a 7-point Likert scale to measure the proposed constructs in line with theoretical interpretations. Therefore, we transformed the raw Likert scale data into fuzzy set scores. Following previous research, we established calibration anchors with the full membership score at 6 (95%), the crossover point score at 4.5 (50%), and the full non-membership score at 3 (5%) [61,63]. After data calibration, a truth table was constructed with a frequency threshold of 5, and combinations with frequencies below 5 were removed [62,64]. Previous research shows that the consistency level should be greater than 0.75 [62–64]. As a result, this study used a higher consistency level (vs. the recommended threshold) to conduct the analysis. This study evaluated the findings based on the two following models:

Model 1: EMC (Presence) = *f* (PRS, INT, CEU, CBT, ENG, CSP)

Model 2: ~EMC (Negation) = *f* (~PRS, ~INT, ~CEU, ~CBT, ~ENG, ~CSP)

#### 4.3.2. Necessary conditions analysis.

A necessary condition analysis was undertaken to determine which conditions are required to generate a strong emotional connection. The proposed fsQCA guidelines describe a condition as necessary if its consistency exceeds 0.90 [65,66]. Table 8 demonstrates that both the presence and the negated condition are insufficient to meet the required threshold. Despite a high consistency of 0.818, engagement remained below the established threshold. These findings suggest that no single condition is necessary for developing emotional connections; instead, various combinations of conditions may affect the emotional connection of tourists. These findings support the configurational mechanism that underlies fsQCA [67]. The findings serve as a roadmap for further sufficiency analysis, which explores how various combinations of conditions foster emotional connection.

**Table 8:** Necessary conditions analysis.

Outcome: EMC		
**Condition Tested**	**Consistency**	**Coverage**
PRS	0.763	0.777
~PRS	0.493	0.626
INT	0.767	0.779
~INT	0.505	0.645
CEU	0.701	0.796
~CEU	0.579	0.651
CBT	0.741	0.835
~CBT	0.549	0.623
ENG	0.818	0.806
~ENG	0.463	0.614
CSP	0.745	0.837

Source: “Authors’ own work”

#### 4.3.3. Truth table and configural outcomes.

After conducting the necessary condition analysis, a truth table was developed to identify the mechanisms for generating configurations that promote emotional connection (see Table 9). In line with existing literature and considering the larger sample size (n = 690), a frequency threshold of five cases and a consistency threshold of 0.900 were used to retain configurations that are both empirically meaningful and theoretically relevant [66,67]. Following that, a sufficiency analysis was conducted to identify various configurations that exhibit a strong association with emotional connection. We analyzed both parsimonious and intermediate solutions to differentiate between core and peripheral conditions. The existing literature indicates that conditions present in both the parsimonious and intermediate solutions are categorized as core conditions, while those found solely in the intermediate solution are categorized as peripheral conditions [62,66,67]. The results indicate that parsimonious solutions, including PRS, CEU, and ENG, are identified as core conditions that serve as key factors of emotional connection, whereas INT, CBT, and CSP have been classified as peripheral conditions, suggesting that their contributions differ across various alternative configurations.

**Table 9:** Truth table.

PRS	INT	CEU	CBT	ENG	CSP	Number	EMC	Raw consist.	PRI consist.	SYM consist
1	1	0	1	1	0	5	1	0.972	0.900	0.900
1	0	1	1	1	1	8	1	0.969	0.892	0.893
0	1	0	1	1	0	5	1	0.967	0.861	0.861
0	1	1	0	1	0	6	1	0.964	0.852	0.857
1	1	0	1	1	1	8	1	0.963	0.891	0.891
1	1	1	1	1	0	10	1	0.963	0.874	0.876
0	1	1	1	1	1	6	1	0.963	0.875	0.887
1	1	1	1	1	1	21	1	0.957	0.882	0.887
1	1	1	0	1	1	6	1	0.956	0.855	0.857
1	1	1	0	1	0	5	1	0.949	0.807	0.807
1	0	1	0	0	0	6	1	0.924	0.626	0.626
1	1	1	0	0	0	7	1	0.909	0.633	0.640
0	0	0	0	1	0	5	1	0.900	0.569	0.569
0	0	0	0	0	0	98	0	0.501	0.091	0.093

Source: “Authors’ own work”

Table 10 shows the intermediate solutions, revealing various configurations that foster a strong emotional connection. Some configurations greatly strengthen tourists’ emotional connection toward AI chatbots. For example, PRS*INT*CBT*ENG demonstrated the optimum raw coverage = 0.513 and consistency = 0.940, indicating that the combined effect of personalization, interactivity, chatbot trust, and engagement contributes to emotional connection. The PRS*INT*CEU*ENG configuration yields a raw coverage = 0.487 and consistency = 0.925, suggesting that the interplay of personalization, interactivity, chatbot ease of use, and engagement effectively fosters emotional connection. The PRS*CEU*CBT*ENG*CSP configuration shows a raw coverage = 0.469, and consistency = 0.956, highlighting the complementary role of chatbot trust and social presence as peripheral conditions in addition to personalization, chatbot ease of use, and engagement as core conditions, in strengthening emotional connections. The INT*CEU*CBT*ENG*CSP configuration has a raw coverage of 0.473 and a consistency of 0.949, highlighting the roles of interactivity, chatbot trust, and social presence as peripheral conditions, along with chatbot ease of use and engagement, in promoting emotional connection. These configurations illustrate the principle of equifinality by showcasing various combinations of chatbot determinants and relational mechanisms that foster emotional connection [66,67]. The overall solution coverage (0.687) demonstrates that reported configurations offer substantial evidence to foster emotional connection, while the overall solution consistency (0.879) exceeds the minimum threshold of 0.75, which increases the reliability and validity of the discovered configurations [66]. These findings collectively demonstrate that both core and peripheral configurations significantly enhance emotional connection. Particularly, engagement emerged as a core condition in all configurations, underscoring its vital role in strengthening tourists’ emotional connection with AI chatbots.

**Table 10:** Configuration results.

Configural Combination	PRS	INT	CEU	CBT	ENG	CSP	Raw coverage	Unique coverage	Consistency
PRS*INT*CEU*ENG	⬤	●	⬤		⬤		0.487	0.019	0.925
PRS*INT*CBT*ENG	⬤	●		●	⬤		0.513	0.027	0.940
PRS*CEU* ~ CBT* ~ ENG* ~ CSP	⬤		⬤	⮾	⮾	⮾	0.243	0.018	0.899
INT*CEU* ~ CBT*ENG* ~ CSP		●	⬤	⮾	⬤	⮾	0.321	0.008	0.942
INT* ~ CEU*CBT*ENG* ~ CSP		●	⮾	●	⬤	⮾	0.336	0.007	0.960
PRS*CEU*CBT*ENG*CSP	⬤		⬤	●	⬤	●	0.469	0.033	0.956
INT*CEU*CBT*ENG*CSP		●	⬤	●	⬤	●	0.473	0.02	0.949
~PRS* ~ INT* ~ CEU* ~ CBT*ENG* ~ CSP	⮾	⮾	⮾	⮾	⬤	⮾	0.196	0.015	0.900

Note: Solution coverage: 0.687; Solution consistency: 0.879; PRS = Personalization; INT = Interactivity; CEU = Chatbot ease of use; CBT = Chatbot trust; ENG ● = Engagement; CSP = Chatbot social presence; ⬤ = Core presence; ● = Peripheral presence; ⮾ = Peripheral negation, Source: “Authors’ own work”

## 5. Discussions

This study reveals exciting insights and contributes meaningfully to theory and practice. First, the findings showed that AI-driven chatbot determinants, such as personalization, interactivity, and chatbot ease of use, contribute to building chatbot trust. Nowadays, personalization, interactivity, and chatbot ease of use are important factors in relationship marketing, offering relational benefits, such as quick help, responsiveness, and easy interactions, building chatbot trust in the travel and tourism industry. Previous research primarily focused on communication service quality, customer satisfaction, service co-creation, and chatbot usage intentions [11,14,16,27,28]. However, previous research lacks insight into the impacts of AI-driven chatbots on psychological mechanisms, such as chatbot trust. This study discovers that AI chatbot determinants are highly relevant to developing trust in chatbots in the Chinese travel industry.

Second, the findings indicate that the direct and indirect (mediating) effects of chatbot trust have become critical for improving engagement and emotional connection in the Chinese travel industry. Chatbot trust can be enhanced by understanding user preferences and by offering quick, accurate, real-time information that can foster engagement [29]. As chatbot trust increases, emotional ties with customers deepen, which can increase positive WOM and customer retention [1]. Indirect mediation signifies the criticality of chatbot trust in the Chinese travel industry. Previous research demonstrated that ChatGPT trust plays an essential role in the relationships between ChatGPT antecedents, such as perceived value and social influence, and user ChatGPT acceptance behavior [68]. This study contributes new knowledge to the literature by translating AI-chatbot determinants into engagement and emotional connection via chatbot trust.

Third, the findings demonstrate the direct and indirect (mediating) effects of engagement to enhance emotional connection in the Chinese travel ecosystem. The findings revealed that higher customer engagement leads to emotional relationships. Likewise, when customers interact more frequently with trusted chatbots, they develop emotional bonds with AI chatbots. Engagement is the pillar for strengthening customer relationships. For example, AI chatbot communication increases engagement, which positively impacts customer satisfaction [36]. Previous research indicates that engagement translates psychological relationships through trust [35]. Therefore, leveraging chatbot trust advantages, Chinese OTAs should improve engagement to foster emotional connections with customers.

Finally, the findings indicate that chatbot social presence significantly moderates the relationships between chatbot trust and engagement but has no effect on the relationships between chatbot trust and emotional connection. The findings are supported by social presence theory, suggesting that users feel a greater sense of presence when they are interacting with systems that reveal social interactivity or human-like attributes, which fosters engagement [37]. Research shows that a strong social presence enhances social interactions, which increases trust and improves engagement with the system [38]. Trust is a basic factor in human-machine communication, which boosts engagement [29]. Thus, when Chinese customers perceive a chatbot as a social entity capable of interacting with others, it enhances their trust and engagement.

Contrary to expectations, the interactive effects of chatbot social presence and chatbot trust did not impact emotional connection. It indicates that emotional connections may require deeper trust and affective relationships to develop long-lasting relationships [18,23]. The findings demonstrate that an emotional connection with tourists can be developed through chatbot trust rather than by focusing on AI chatbots’ perceived social presence. According to social presence theory, people’s interpretations of social signals are influenced by their interactions with others and can be categorized as social or functional. Social signals have little impact on consumer responses when interactions are purely instrumental [69]. Therefore, users may not value the social features of chatbot conversations as much as the practical features of the OTA platform, such as trustworthiness, safety, and accuracy. In addition, chatbot anthropomorphic attributes can boost social presence and connections, but users are aware that these systems are automated [70]. Therefore, social presence may have a lesser impact on the formation of emotional relationships with others.

The fsQCA findings revealed several unique configurations that lead to enhanced emotional outcomes in chatbot-mediated service interactions. First, the finding indicates that AI chatbot attributes, such as personalization, interactivity, and ease of use, improve customer emotional connections. These findings contribute to relationship marketing, commitment-trust, and social presence theories, indicating that AI chatbot attributes improve relational outcomes, specifically emotional connection [18,24,40]. Second, the findings revealed that tourists’ trust in AI chatbots is crucial to developing an emotional connection. These findings aligned with relationship marketing theory and commitment-trust theory, suggesting that trust is a central variable that enhances long-term relationships [18,23]. Third, the findings showed that engagement is a powerful driver of emotional relational outcomes. The findings are aligned with the RMT, indicating that engagement motivates customers to improve their relationships with firms [24]. Therefore, the engagement mechanism plays an important role in elevating customer emotional connections in the Chinese travel industry.

### 5.1. *Theoretical contributions*

This study significantly contributes to relationship marketing theory (RMT), commitment-trust theory (CTT), and social presence theory in the context of AI chatbots in China’s travel industry. Traditional RMT emphasized human-to-human interactions in enhancing trust [18,24]. Our findings extend these interactions from machine-to-human to improve individuals’ trust in machines. For example, AI chatbot determinants, including personalization, interactivity, and chatbot ease of use, can help develop trust. The findings derived from the perspectives of relationship marketing, trust–commitment, and social presence theories enhance the relationship between AI chatbots and tourists within the context of China’s emerging travel industry [18,23,40]. The findings strengthen CTT in the AI context, indicating that chatbot trust can provide relational support related to human-machine interactions. The findings also contribute to RMT by fostering AI engagement and emotional connections, supporting AI-traveler relationships, and integrating another layer to prevailing relationship theories. The findings contribute to CTT and RMT [18,23,24] by indicating that engagement is a relational driver that enhances emotional connections toward AI chatbots. In addition, engagement as a positive mediator bridges the gap between chatbot trust and emotional connection in the AI context. The findings contribute to the social presence theory in the context of the burgeoning domain of human-AI interactions [37,40]. Social presence theory offers a robust framework for predicting chatbot social presence and its effects on cognitive and behavioral outcomes (i.e., engagement).

### 5.2. *Managerial implications*

This study offers several guidelines for digital marketing managers. First, travel platforms can improve tourist trust by providing customized AI chatbot services that enable effortless interactions with minimal input and smooth navigation. They can implement deep personalization via tourist data and real-time behavioral tendencies. Improved interactions should reflect consciousness, awareness, and empathy, aligning with travelers’ expectations through harmonious, respectful language. In addition, measuring relational key performance indicators (KPIs) like trust and commitment is essential to sustain relational value. Second, the findings showed that chatbot trust is highly relevant in the Chinese travel industry. All online travel agencies (OTAs) should enhance trust by ensuring data privacy, transparency, consistency, and response accuracy, which will help improve interactions and foster emotional ties with customers. Third, the findings showed that along with chatbot trust, OTAs should plan campaigns to enhance customer engagement, as it leads to emotional connections. In addition, managers must prioritize building emotional relationships with customers, which can lead to positive WOM, loyalty, and advocacy. Instead of focusing on chatbot functions, managers should emphasize chatbots as relational agents to gain a competitive advantage in China’s dynamic OTA market. Finally, considering the significant effects of chatbot social presence, OTAs must integrate it with consistent personalized narratives, service quality, and cultural relevance to promote deeper engagement. Likewise, the dual-layered strategy, such as social presence and trust, can elevate customer-chatbot relational dynamics in the Chinese AI-driven travel market.

### 5.3. *Limitations and future research agenda*

This study has several limitations. For example, this study was conducted exclusively in the context of China. Future research may evaluate the role of AI-driven chatbots in developing relational outcomes in other countries, specifically in Europe, or conduct cross-cultural research to deepen insights into AI-driven chatbots. This study used a cross-sectional methodology, with all data taken from a single source. While proposed relationships are theoretically valid and supported by empirical evidence, the cross-sectional design is insufficient to facilitate definitive causal findings. Future research should employ longitudinal or experimental methods to provide additional evidence regarding the causal relationships among chatbot determinants, trust, engagement, and emotional connection. This study integrated a single mediator (i.e., chatbot trust) into the AI relational framework. Future research may explore other mediators, such as service or relationship quality. This study incorporated a single moderator (i.e., chatbot social presence); future research may incorporate other relational variables, such as attachment or evangelism, to strengthen the relationship theories. This study employed purposive sampling and primarily focused on digitally oriented respondents aged 18–33 years old, which may limit the generalizability of the findings. Future research can utilize other sampling techniques, such as random sampling, and gather data from a diverse range of age groups to generalize the findings to the entire population of Chinese tourists. Finally, this study did not consider control variables. Researchers may integrate control variables, such as gender, age, income, and education, into the proposed framework to reveal more compelling insights.
